# Physio-biochemical responses and crop performance analysis in chickpea upon botanical priming

**DOI:** 10.1038/s41598-024-59878-8

**Published:** 2024-04-23

**Authors:** Kamini Kaushal, Kumari Rajani, Ravi Ranjan Kumar, Tushar Ranjan, Anand Kumar, M. Feza Ahmad, Vikash Kumar, Vinod Kumar, Aman Kumar

**Affiliations:** 1 Department of Seed Science and Technology, Bihar Agricultural University, Sabour, Bhagalpur, 813210 India; 2 Department of Molecular Biology and Genetic Engineering, Bihar Agricultural University, Sabour, Bhagalpur, 813210 India; 3 Department of Plant Breeding and Genetics, Bihar Agricultural University, Sabour, Bhagalpur, 813210 India; 4 Bhola Paswan Shastri Agricultural College, Bihar Agricultural University, Sabour, Purnea, 854302 India; 5 Department of Soil Science and Agricultural Chemistry, Bihar Agricultural University, Sabour, Bhagalpur, 813210 India; 6 Division of Seed Science and Technology, Indian Agricultural Research Institute, Pusa, New Delhi, 110012 India

**Keywords:** Botanical priming, Turmeric, Dehydrogenase, Globulin, Vigour index, SDS PAGE, Biotechnology, Plant sciences

## Abstract

Chickpea is a highly nutritious protein-rich source and one of the major crops to alleviate global malnutrition, but poor seed quality affects its productivity. Seed quality is essential for better crop establishment and higher yields, particularly in the uncertain climate change. The present study investigated the impact of botanical priming versus hydropriming and bavistin seed treatment on chickpea seeds. A detailed physiological (germination percentage, root and shoot length, vigour index) and biochemical (amylase, protease, dehydrogenase, phytase, and lipid peroxidation) analysis was carried out in order to assess the effect of priming treatments. Turmeric-primed seeds showed better germination rate (94.5%), seedling length, enzyme activity, and lower malondialdehyde (MDA) content. Sodium dodecyl-sulfate polyacrylamide gel electrophoresis (SDS-PAGE) analysis revealed the expression of minor polypeptides of albumin and globulin in the primed seeds. Moreover, field experiments indicated increased crop growth, vigour, days to 50% flowering, yield and its attributing traits in turmeric-primed seeds. Botanical priming can increase chickpea yield by up to 16% over the control group. This low-cost and eco-friendly technique enhances seed and crop performance, making it a powerful tool for augmenting chickpea growth. Therefore, chickpea growers must adopt botanical priming techniques to enhance the quality of seed and crop performance. Moreover, this approach is environmentally sustainable and can help conserve natural resources in the long term. Therefore, this new approach must be widely adopted across the agricultural industry to ensure sustainable and profitable farming practices.

## Introduction

Global climate change and water scarcity generally affect crop performance from germination and eventually reduce grain yield and quality with losses of up to 40–50% in crop productivity. Chickpea, a cool-season legume, are an excellent source of protein (18–20%) and the minerals phosphorus, calcium, magnesium, iron, and zinc that play a significant role in achieving global food security^1^. Over 90% of the world's chickpeas are grown in arid and semi-arid regions, making them vulnerable to various stresses^2,3^. Even though it is considered the candidate crop for rainfed conditions, the uncertain climatic condition adversely affects the crop's performance and makes it vulnerable to several biotic and abiotic stresses^4^.

Successful seedling and crop establishment are critical to higher crop production^5^. Poor seedling establishment is one of the most significant barriers primarily caused by subpar seed quality^6^. The improper storage is the primary factor for quick loss of seed vigour, which ultimately affects germination and crop performance. Moreover, crop germination due to adverse climatic conditions and delayed sowing affects production and productivity. Seed quality enhancement techniques provide a comprehensive solution to unlock the full genetic potential of seeds. Seed quality enhancement, a pre-sowing treatment, enhances germination and crop vigour during early planting and contributes to a higher crop yield^7^.

Seed priming, a technique in seed enhancement, involves the initial absorption of water by seeds to initiate the early stages of germination. However, this absorption is insufficient for radical emergence, and the seeds return to their original moisture content^8^. During the priming process, biochemical changes occur, including activating enzymes, producing compounds that promote growth, metabolizing molecules that inhibit germination, and repairing damaged cells^9^. The imbibition of seeds triggers a cascade of metabolic processes, such as the activation of hydrolytic enzymes (such as amylase, protease, lipase, dehydrogenase, and phytase), which lead to the hydrolysis of stored starch, lipids, proteins, polyphosphates, and other storage materials, converting them into simpler forms that readily absorbed by the embryo and ultimately influencing seed vigour^10^. Alpha-amylase breaks down starch into sugars for the developing embryo^11^. Proteolytic enzymes use seed storage proteins^12^, while dehydrogenase catalyzes the stored products during the anaerobic phase of seed germination^13^.

Phytase is an enzyme that unequivocally converts phytate into inositol/phosphoric acid, making a remarkable contribution to seed germination and growth^14^. On the other hand, lipid peroxidation is an unequivocally detrimental process that destabilizes the membrane and degrades proteins, leading to inevitable cell death, which hinders the capacity for ionic transport^15^. Similarly, the hydrolysis of seed storage proteins such as globulin and albumin unequivocally generates free amino acids, essential for germination and seed vigour^16^.

Over the past few decades, there has been a significant increase in the utilization of non-hazardous chemicals and fertilizers as alternatives to enhance crop productivity in the agriculture system. Among these alternatives, priming has emerged as a highly effective method to tackle this issue. Numerous efficacious priming agents, such as salts, polyamines, hormones, compatible solutes, and aqueous plant extracts, have been documented by various researchers^17^. Botanical priming, a priming technique that employs plant extracts as its agents, has proven particularly advantageous. This botanical priming method can stimulate metabolic processes, is non-toxic and environmentally friendly, and has excellent potential to control pathogenic microorganisms.

There are few reports on using turmeric rhizome and neem seed extracts for aphid control, plant growth, and crop yield. However, the effect of leaf extracts on seed physiology and plant growth in pulse crops has yet to be studied. No studies have investigated globulin and albumin expression in chickpeas during priming and germination at different durations. In the present investigation, we hypothesized that, first, the effect of turmeric and neem leaf extracts as seed priming agents positively influences physiological parameters and enhances seed vigour in chickpeas. Second, the improved enzymatic activities during botanical priming lead to increased seed germination and growth. Third, priming also increases the expression of different seed storage proteins compared to non-primed conditions. Our objective was to investigate the effect of botanical priming on physiological, biochemical and yield in chickpeas, for which we performed comprehensive laboratory and field experiments.

## Results

### Effect of botanical priming on seed germination and seedling characteristics

The present study was carried out to analyze the impact of seed priming with botanicals (turmeric, neem) on the physiological parameters of chickpeas and compare it with conventional hydropriming and fungicide seed treatment. In the present investigation, physiological parameters, viz germination percentage and seedling characteristics, were significantly increased by priming the seeds with botanicals, as shown in Table 1. The botanical primed seed had substantially higher seed germination and seedling growth than the control. Seeds primed with turmeric and neem leaves aqueous extract had higher germination and seedling growth followed by hydro priming. The results demonstrate that turmeric had a higher germination percentage (94.5%) than control (82.5%), indicating that botanical priming successfully enhanced the germination rate by 15% over control. Seedling characteristics, including root length, shoot length, seedling length, seedling dry weight and vigour indices, were significantly similarly affected by botanical priming. Table 1 shows marked enhancement in seedling length by 29%, vigour index Ι by 42% and vigour index II by 63% were observed in turmeric primed seed over control. Seed physiological parameters, such as seedling length, vigour index I, and vigour index II, were significantly affected by turmeric priming compared to other treatments such as neem and hydropriming. As illustrated in Table 1 and supplementary information file 1, a marked enhancement in seedling length by 8.6%, vigour index I by 30.4%, and vigour index II by 21.8% was observed in turmeric-primed seeds compared to those treated with neem

**Table 1:** The impact of different priming agents on germination percentage, root length, shoot length, seedling length, dry weight and vigor indices was estimated on the 8th day of germination.

Treatments	Germination (%)	Root length (cm)	Shoot length (cm)	Seedling length (cm)	Dry weight (g)	Vigour Index Ι	Vigour Index ΙΙ
T_1_	82.5^b^	17.5^a^	10.73^c^	24.77^c^	0.41^c^	2050.14^d^	33.24^c^
T_2_	83.5^b^	15.07^c^	10.97^c^	26.99^c^	0.43^c^	2226.8^c^	35.52^c^
T_3_	94.5^a^	18.16^a^	13.77^a^	31.93^a^	0.6^a^	2905.71^a^	54.13^a^
T_4_	86.75^b^	16.02^b^	13.38^a^	29.4^b^	0.51^b^	2226.8^c^	44.44^b^
T_5_	86.75^b^	14.04^d^	12.02^b^	26.06^c^	0.49^b^	2549.21^b^	42.68^b^
S.E.M	2.02	0.27	0.32	0.8	0.01	57.97	1.24
C.D. 5%	6.1	0.8	0.96	2.41	0.04	174.75	3.73
C.V	4.66	3.28	5.22	5.74	5.28	4.85	5.89

Data presented in a column are means of four replicates, where each replicate of the treatment contains 100 seeds. Means in each column, followed by different lower-case alphabets, are significantly different at the 0.05% probability level using the Post Hoc test. Where T_1_ = No priming (control), T_2_ = Conventional practice (Bavistin@2 g/kg), T_3_ = Turmeric leaf priming, T_4_ = Neem leaf priming, T_5_ = On-farm priming (Hydropriming).

### Biochemical changes upon botanical priming

**Figure 1:**
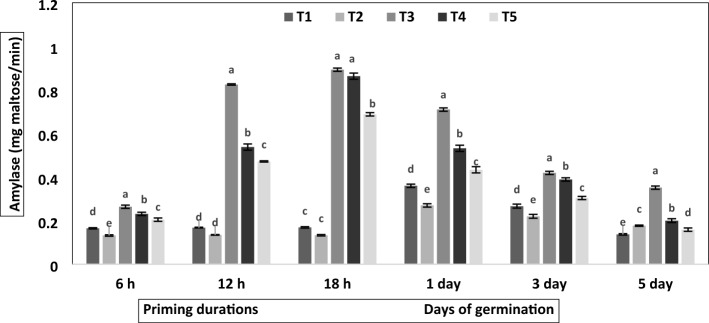
Effect of different priming agents on amylase activity during 6 h, 12 h and 18 h duration of priming and 1st, 3rd, and 5th days of germination in primed and non-primed seed of chickpea variety PG-186. Data presented are means of four replicates with standard deviation. Different letters indicate significant differences within each treatment by Post Hoc test at *P* = 0.05 levels.T_1_ = No priming (control), T_2_ = Conventional practice (Bavistin@2 g/kg), T_3_ = Turmeric leaf priming, T_4_ = Neem leaf priming, T_5_ = On-farm priming (Hydropriming).

**Figure 2:**
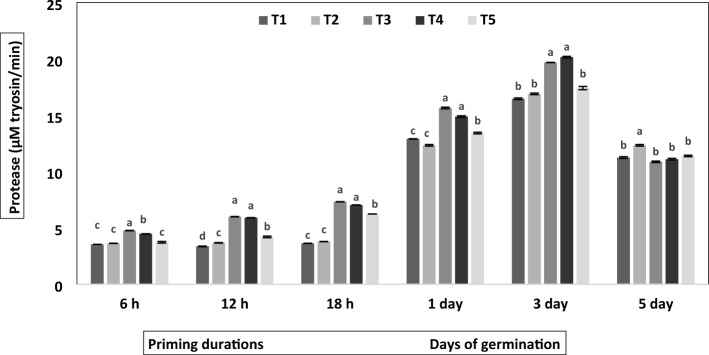
Effect of different priming agents on protease activity during 6 h, 12 h and 18 h duration of priming and 1st, 3rd, and 5th days of germination in the primed and non-primed seed of chickpea variety PG-186. Error bars are the representative of the standard deviation of four replicates. Different letters indicate significant differences by Post Hoc test at *P* = 0.05 levels within the treatment. T_1_ = No priming (control), T_2_ = Conventional practice (Bavistin@2 g/kg), T_3_ = Turmeric leaf priming, T_4_ = Neem leaf priming, T_5_ = On-farm priming (Hydropriming).

**Figure 3:**
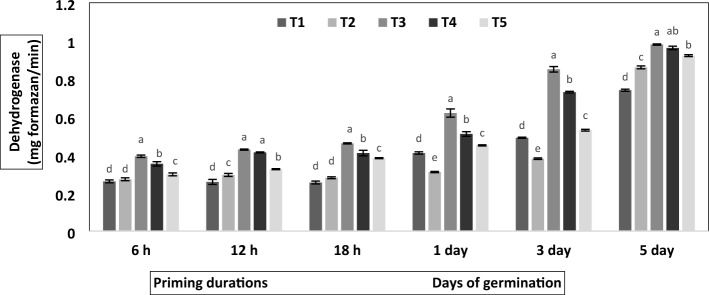
Effect of botanical priming on dehydrogenase activity during 6 h, 12 h and 18 h duration of priming and 1st, 3rd, the 5th days of germination in primed and non-primed seed of chickpea variety PG-186.vertical bar presented are means of four replicates with standard deviation. Different letters indicate significant differences within each treatment by Post Hoc test at *P* = 0.05 levels. T_1_ = No priming (control), T_2_ = Conventional practice (Bavistin@2 g/kg), T_3_ = Turmeric leaf priming, T_4_ = Neem leaf priming, T_5_ = On-farm priming (Hydropriming).

**Figure 4:**
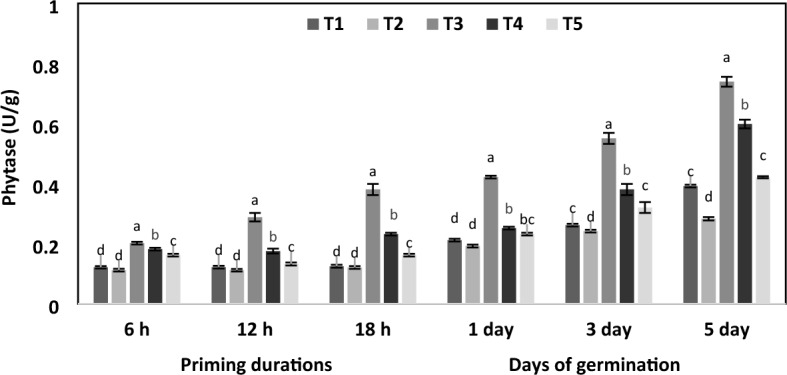
Effect of different priming agents on phytase activity during 6 h, 12 h and 18 h duration of priming and 1st, 3rd, and 5th days of germination in primed and non-primed seed of chickpea variety PG-186. Error bars are the representative of the standard deviation of four replicates. Different letters indicate significant differences within each treatment by Post Hoc test at *P* = 0.05 levels. T_1_ = No priming (control), T_2_ = Conventional practice (Bavistin@2 g/kg), T_3_ = Turmeric leaf priming, T_4_ = Neem leaf priming, T_5_ = On-farm priming (Hydropriming).

**Figure 5:**
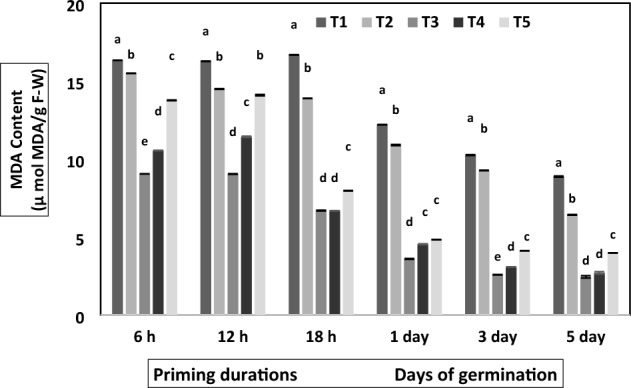
Effect of different priming agents on MDA content during 6 h, 12 h and 18 h duration of priming and 1st, 3rd, and 5th days of germination in primed and non-primed seed of chickpea variety PG-186. Data presented are means of four replicates with the standard deviation. Different letters indicate significant differences within each treatment by Post Hoc test at *P* = 0.05 levels. T_1_ = No priming (control), T_2_ = Conventional practice (Bavistin@2 g/kg), T_3_ = Turmeric leaf priming, T_4_ = Neem leaf priming, T_5_ = On-farm priming (Hydropriming).

The dynamic changes in hydrolytic enzymes and MDA content are shown in Figs. 1, 2, 3, 4, 5 and supplementary information file 2. Figure 1 shows that botanical priming and hydropriming significantly affected amylase activity, which increased with the increase in priming duration. In contrast, the amylase activity was decreased as the germination proceeded. Conversely, the amylase activity was constant at different priming hours in non-primed and bavistin-treated seeds; however, they showed a similar decreasing trend during germination as botanicals primed seed. Compared to non-primed seeds, maximum amylase activity was observed in turmeric-primed seeds, and it was increased to 4.8-fold at 12 h and 5.2-fold at 18 h of priming. The amylase activity was also assessed during germination, and it showed a decreasing trend with the increasing days of germination, and it was highest in turmeric primed seed. The highest amylase activity, 0.71 mg maltose/min, was observed on the first day of germination on the first day, and its activity decreased to 1.7-fold on the third day and 2.0-fold on the fifth day of germination.

Botanical priming significantly affected the protease activity (*P* < 0.05; Fig. 2). The protease activity significantly increased with increasing priming duration and till the third day of germination; after that, on the fifth day of germination, irrespective of treatments, the protease activity decreased. The protease activity was maximum during priming durations and germination days in seeds primed with 1% aqueous extract of turmeric leaves. Its activity increased to 1.3-fold in 6 h, 1.8-fold in 12 h, two fold in 18 h, 1.2-fold on the first day and 3.2-fold on the third day compared to control in turmeric primed seed. On the fifth day, there was a drastic reduction in the protease activity, which decreased by 1.8-fold compared to the third day of the same treatment. A similar pattern was observed in 1% aqueous extract of neem leaves and hydro-priming, whereas activity was constant throughout the priming duration in control and bavistin-treated seed.

In the current study, botanical priming significantly affected dehydrogenase activity (*P* < 0.05; Fig. 3) and differed among treatments. The dehydrogenase activity was significantly increased with priming duration and days of germination. The maximum dehydrogenase activity of 0.98 mg formazan/min was observed on the fifth day of germination in seeds primed with 1% aqueous extract of turmeric leaves. Its activity increased to 1.84-fold in 18 h and 1.3-fold on the fifth day of germination compared to the control.

To confirm the activation of dormant phytase zymogens induced by botanical priming, we investigated the dynamic changes in phytase activity during priming durations and days of germination. As shown in Fig. 4, the phytase activity significantly increased with increasing priming duration and days of germination. The maximum activity was observed in turmeric primed seed during the fifth day of germination, and it was 0.74 µM trypsin/min (1.9-fold increased over control at a particular time). Compared to the control, its activity in turmeric primed seed increased to 1.7-fold in 6 h, 2.4-fold in 12 h, and 2.9-fold in 18 h of priming. The phytase remained active during germination and increased by 2-fold on the first day, 2.1-fold on the third day and 1.9-fold on the fifth day of germination.

Botanically primed seed showed a significant reduction in lipid peroxidation activity, measured in terms of MDA content (µmol MDA/gr F-W). In the present study, as shown in Fig. 5, on increasing the priming duration, the MDA content was not found to change up to 12 h in turmeric primed seed; after that, it decreased sharply at 18 h of priming and during days of germination. Compared to the control, the MDA content decreased to 2.49-fold in 18 h of priming, 3.4-fold on the first day, four fold on the third day, and 3.6-fold on the fifth day of germination and the maximum MDA content was observed in the case of the control seed (T_1_).

### Effects of botanical priming on total protein and the expression of seed storage protein

**Figure 6:**
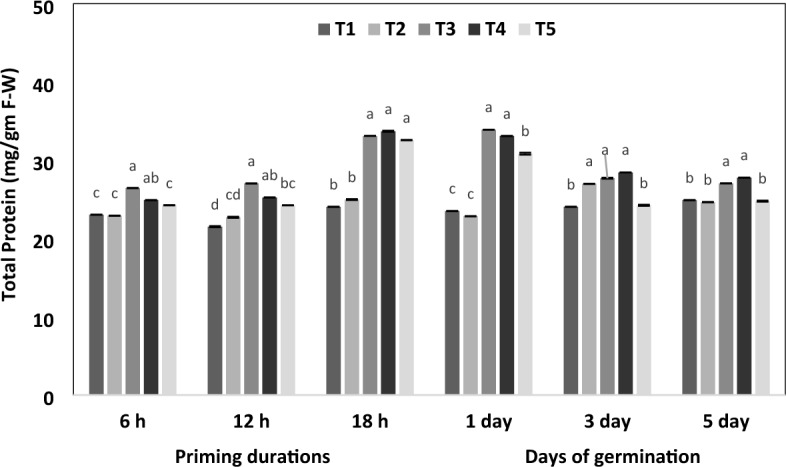
Impact of different priming treatments on total protein content in chickpea during 6 h, 12 h and 18 h duration of priming and 1st, 3rd, and 5th days of germination in primed and non-primed seed of chickpea variety PG-186. Data presented in each column are means of four replicates with standard deviation within treatments. Different letters indicate significant differences by post-hoc test at the *P* = 0.05% level. T_1_ = No priming (control), T_2_ = Conventional practice (Bavistin@2 g/kg), T_3_ = Turmeric leaf priming, T_4_ = Neem leaf priming, T_5_ = On-farm priming (Hydropriming).

**Figure 7:**
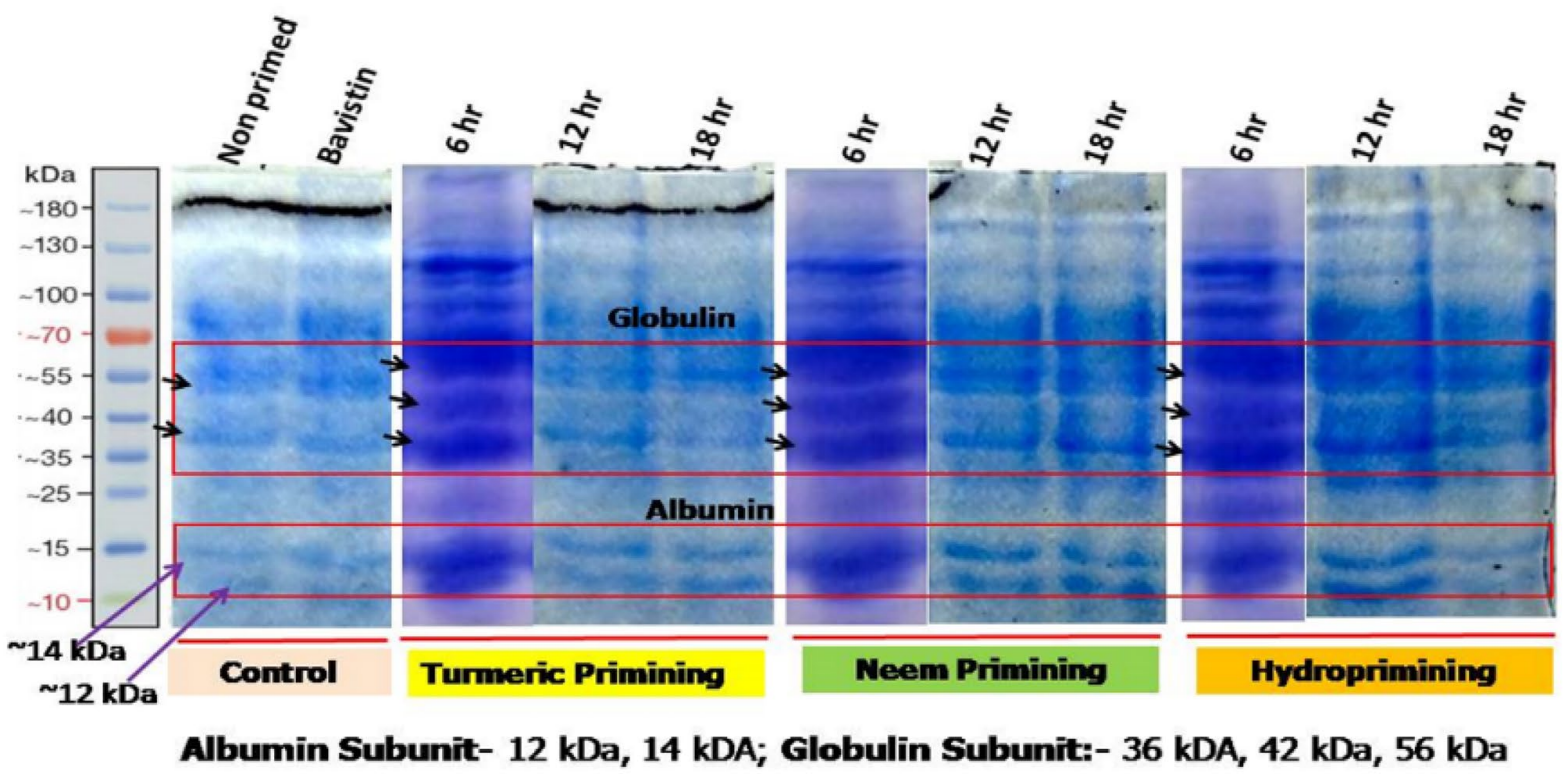
SDS- PAGE (12%) of chickpea seed storage protein extracted and characterized by Osbornes methods using the borate buffer. Shows the effect of different priming treatments on the expression of globulin and albumin subunits during 6,12,18 h of priming.12 kDa and 14 kDa are the subunits of albumin and 36 kDa, 42 kDa and 56 kDa are the globulin subunits. Seed treatments include T_1_ = No priming (control), T_2_ = Conventional practice (Bavistin@2 g/kg), T_3_ = Turmeric leaf priming, T_4_ = Neem leaf priming, T_5_ = On-farm priming (Hydropriming).

The impact of botanical priming on total protein content was significant in our present work; as illustrated in Fig. 6, a considerable upsurge (*p* < 0.05) in protein content was evident during priming hours and days of germination with both turmeric and neem leaf aqueous extract. In this, the total protein content increased with the priming duration up to the first day of germination. However, upon increasing the days of germination, irrespective of the priming material, the total protein decreases significantly from the third to the fifth days. Meanwhile, in the control and bavistin-treated seeds, protein content did not vary much throughout the priming duration and during germination. The total protein content increased 1.4-fold on the first day after that, decreased to 1.1-fold on the third day and one fold on the fifth day of germination compared to the control. Seed storage proteins (SSP) of chickpeas, i.e., globulin and albumin, were fractionalized by the Osborne method^18^ and the molecular weight of the protein was determined using SDS PAGE. Subunits separated from chickpea proteins are shown in Table 2. Supplementary information file 4 and Figure 7 shows that the expression of proteins was higher in primed seeds than in the control. In the present work, the estimated molecular weights of subunits of globulin, i.e., legumin, were (40 kDa, 39 kDa, 26 kDa, 23 kDa), vicillin Mw (50 kDa, 39 kDa, 19 kDa, 15 kDa), and glutelin (58 kDa, 55 kDa, 54 kDa). Three minor bands of 36 kDa, 42 kDa, and 56 kDa, the globulin subunits, were detected in the electrophoregram. Table 2 also reveals that the significant globulin subunits, i.e., 42 kDa and 56 kDa, were present in all primed seeds, whereas control and bavistin polypeptides of 42 kDa proteins were not observed. Our work in SDS-PAGE showed a progressive accumulation of the 11-S globulin during priming. In contrast, a slower accumulation of 11S occurred in control seeds, which resulted in an intense polypeptide of 56 kDa and 42 kDa in primed seeds.

**Table 2:** Effect of botanical priming on the expression of seed storage proteins (globulin and albumin) detected through SDS-PAGE.

Seed storage protein	Molecular weight (kDa)	T_1_	T_2_	Duration of priming (h)								
				T_3_			T_4_			T_5_		
				6	12	18	6	12	18	6	12	18
Globulin	36	+	+	+	+	+	+	+	+	+	+	+
	42	–	–	+	+	+	+	+	+	+	+	+
	56	+	+	+	+	+	+	+	+	+	+	+
Albumin	12	+	+	+	+	+	+	+	+	+	+	+
	14	+	+	+	+	+	+	+	+	+	+	+

Where ( +) and (**–**) signs in each column indicate the presence and absence of polypeptide of globulin and albumin sub unit during 6, 12 and 18 h duration of priming. Seed treatments includeT_1_ = No priming (control), T_2_ = Conventional practice (Bavistin@2 g/kg), T_3_ = Turmeric leaf priming, T_4_ = Neem leaf priming, T_5_ = On-farm priming (Hydropriming).

### Effects of botanical priming on plant establishment, growth and yield of chickpea

An experiment with turmeric leaf extract priming in chickpea was compared with conventional seed treatment (+ control) and non-priming (control) under field conditions and data provided in supplementary information file 3. Table 3 shows that turmeric priming significantly improved field emergence, plant height at different growth stages, days to 50% flowering, and yield attributing characters (Tables 3 and 4). Turmeric priming significantly increased the field emergence by 27%, plant heights at 15 DAS, 45 DAS, and maturity by 5%, 11%, and 4.7%, respectively, compared to the non-priming. About the days to 50% flowering, it was significantly (*p* < 0.05) affected by priming and obtained three days earlier than in the control plant (Table 3). The effect of turmeric leaf priming on yield-attributing characteristics in chickpeas is shown in Table 4, which presents the data on-field performance, where turmeric priming considerably increased the number of pods per plant, biological yield, economic yield, and harvest index compared to the control. In comparison to the control, turmeric priming significantly raised, the number of pods per plant by 31%, the biological yield by 22% and the harvest index by 22.1%.

**Table 3:** Effect of turmeric priming on plant establishment and development.

Treatments	Field emergence (%)	Plant height at 15 days (cm)	Plant height at 30 days (cm)	Plant height at 45 days (cm)	Plant height at maturity (cm)	Days to 50% flowering
T_1_	70.16^c^	12.77^b^	20.34	24.7^b^	56.1^ab^	85.00^a^
T_2_	77.85^b^	12.84^b^	21.31	25.75^ab^	54.88^b^	86.75^a^
T_3_	88.86^a^	13.37^a^	21.4	27.4^a^	58.75^a^	82.5^b^
S.E.M	2.43	0.16	0.59	0.70	1.00	0.77
C.D. 5%	7.36	0.48	NS	2.11	3.03	2.33
C.V	8.70	3.41	7.90	7.58	5.00	2.57

Field emergence %, plant height at 15, 30, 45 days and maturity, days to 50% flowering were observed. The field experiment was conducted in RBD, and the data presented in each column is the means of 8 replicates (n = 8). Means in each column, followed by different lower-case alphabets, significantly differ at the 0.05% probability level using the Post Hoc test.T_1_ = No priming (control), T_2_ = Conventional practice (Bavistin@2 g/kg), T_3_ = Turmeric leaf priming.

**Table 4:** Effect of priming on yield attributing characters of chickpea variety PG-186 on number of pods/plant, seed weight, biological yield, yield kg/ha, economic yield and harvest index.

Treatments	No. of Pods/plant	100 Seed weight (g)	Biological yield (g)	Yield (kg/ha)	Harvest index
T_1_	84.38^b^	17.01	43.28^b^	2007.14^b^	53.6^b^
T_2_	85.00^b^	16.96	48.39^ab^	2039.62^b^	51.51^b^
T_3_	110.25^a^	18.73	52.73^a^	2324.87^a^	65.47^a^
S.E.M	4.20	0.85	1.95	68.61	2.69
C.D. 5%	12.73	NS	5.93	208.11	8.15
C.V	12.73	13.72	11.48	9.14	13.36

Data in each column are means of 8 replicates; different lower-case alphabet show significant levels of difference at 0.05% probability level. T_1_ = No priming (control), T_2_ = Conventional practice (Bavistin@2 g/kg), T_3_ = Turmeric leaf priming.

## Discussion

Seed germination is an intricate process involving several physiological and biochemical changes modulated by several biochemical enzymes, and seed priming has been proven to alleviate the adverse effects of any stress during early seedling establishment. The present study suggested that seeds primed with botanicals accelerated seed germination rate and significantly enhanced seed vigour, as indicated by longer root lengths, shoot lengths, seedling length and seedling dry weight compared with the control (Table 1). Our findings are similar to previous results of botanical priming on black gram using neem and prosopus, demonstrating a significant increase in standard germination, shoot length, root length, seedling length and vigour^19^. Various physiological, biochemical, and molecular changes occurred during seed priming, contributing to improved germination rate and seedling vigour under various environmental conditions^20^. The increase in seedling characteristics was due to enhanced enzyme activity from bioactive substances like curcumin and phenols in the turmeric leaf extract. Similar results were reported in greengram^21^, clusterbean^22^, *Vigna sinensis*^23^, maize^23^ and wheat^24^. The increase in dry weight with botanical treatment may be due to the faster growth and development of seedlings and the hike in vigour index^25^. The high vigour index in botanically primed seeds may be due to growth-promoting compounds and secondary metabolites translocated during seedling growth. Priming improves chickpea starch metabolism by increasing amylase content during germination (Fig. 1). Similar results were obtained by Mukasa and coworkers in sugar beets^26^, where the level of amylase activity in primed sugar beets was 1.9 to 11.5 times higher than in the control group. In similar work on rice, seed priming increases the α-amylase activity and total soluble sugar content, resulting in a higher starch degradation process under chilling stress^27^.

Seed priming induced primary memory, which activates pre-germinative metabolism in seed that triggers gibberellin biosynthesis, antioxidants^28,29^, protein synthesis^28^, amylase and protease activation (Fig. 2), which helps in radical protrusion and enhances the antioxidant defense system against DNA damage^30^. Proteins stored in the seed are utilized during germination to provide amino acids and amides for the embryo's development. Proteases play a crucial role in protein degradation during the maturation process. Protease activity in chickpea declines after the 5th day of germination due to accelerated enzyme degradation. During Initial germination phase, facilitates the mobilization of stored proteins into amino acids and peptides for seedling growth. As the seedling develops and transitions to active growth, the demand for stored proteins decreases; henceforth, the decline in activity in proteases is observed. Protease activity was found highest in primed seeds mainly due to botanical priming (Fig. 2), which showed improved nitrogen metabolism in primed seeds, as reported in the pearl millet^31^. In our investigation, increasing priming duration led to enhanced protease activity (Fig. 2); similar results were obtained in beans where the proteolytic enzyme activity increased during the first seven days of seed germination^32^. Dehydrogenase enzymes are essential components of the electron transport chain, facilitating the transport of electrons and ATP production^33^, and their activity is considered a positive biomarker for testing seed viability and vigour^34^. The present study observed a relatively high amount of dehydrogenase after priming. Similar trends were reported in cucumber^35^ and cowpea^36^, suggesting the role of priming in accelerating dehydrogenase activity.

Seeds store phytic acid (phytate), the phosphorous storage form in the plants^37^, which can bind with essential cations like calcium, magnesium, and zinc, reducing their availability for digestion^38^. However, phytic acid is broken down by the enzyme phytase during germination, releasing cations, phosphates, and inositol utilized by the seedlings^39^. Priming significantly increased the activity of the phytase enzyme (Fig. 4); this may result from the de novo synthesis of phytase during germination^40^. A similar trend was reported for germinating rice^41^, lupin^42^, barley^40^ and soybean^43^. A maximum of seven fold increase in phytase activity was observed on the 10th day of germination in rice^41^. Significant differences in the phytase activity of wheat, rye, barley, and oats grains were observed, with rye grains showing the highest activity and oats being the lowest. After four days, wheat, barley, and oat activities increased approximately 4.5, 6, and nine fold, respectively, and rye activities increased approximately 2.5-fold after three days of germination^44^.

Seed priming significantly decreased the rate of lipid peroxidation in terms of MDA content (Fig. 5). MDA content was reduced after priming due to increased antioxidant enzyme activity^45^. In similar work, malondialdehyde content was 9% lower in primed pea seeds at 42 h of germination against unprimed seeds^46^. This decreased level of MDA indicated reduced lipid peroxidation, which helps maintain the integrity of the membrane in primed seeds^47^. In our finding, the protein content was significantly affected upon botanical priming, where the protein content was increased during priming and subsequently decreased during germination (Fig. 6). This was in accordance with the reports documented from black chickpea primed with MgO nanoparticles^28^. After priming, two minor bands of 12 kDa and 14 kDa subunits of albumin were identified (Fig. 7); our findings are similar to previous research^48^, where it was reported that chickpea 2S albumin (∼20 kDa) is composed of two polypeptides of 10 and 12 kDa. In similar findings, it was hypothesized that the extra peptide could be a peptide of 2S albumin with Mw 4–10 kDa^49^. Similar trends were reported in cucumber^35^ and black beans^50^. The probable reason for the extra one band in all treated seeds is priming, resulting in the synthesis of lost proteins and some new ones.

**Figure 8:**
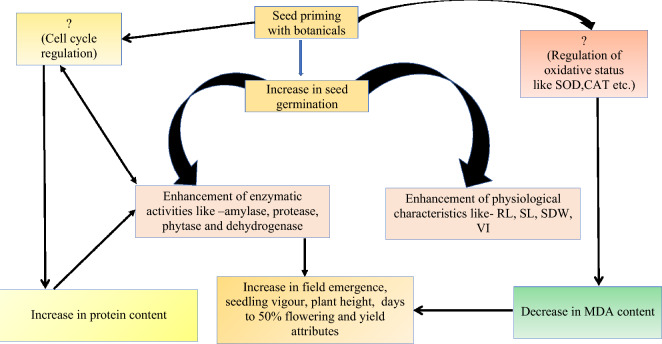
Possible mechanism upon botanical seed priming in chickpea.

Chickpea seeds primed with aqueous leaf extract of turmeric had higher field emergence and plant heights during early establishment and at maturity than the control (Table 3). In the present study, turmeric leaf extract priming improved the seedling growth attributes by triggering the biosynthesis of nucleic acid, proteins, and hydrolytic enzymes and consequentially enhanced the cell division, cell enlargement, and metabolic activity and increased the photosynthetic process of the plant, resulting in increased uptake of more nutrients by efficient and more robust roots (Fig. 8). In similar works on nanoparticle priming, AgNPs accumulated in the seeds might activate the metabolic events vital for seed germination and seedling growth^51^. Significant differences were observed in the number of pods, seed yield, and harvest index in turmeric primed seed over control (Table 4). The results are in accordance with the studies on maize, where seeds primed with prosopis and moringa leaf extracts led to higher seed yield and yield-related parameters^52^. Physiologically active substances in the turmeric leaf may have activated the embryo growth, resulting in early seedling emergence from the soil. The early growth of roots is vital for establishing a robust and efficient root system, which contributes to the development of higher seedling vigour. The elasticity of the cell walls plays a significant role in ensuring effective water absorption, which is essential for the healthy growth of plants. As a result, the seedlings are better equipped to cope with the challenges of their environment, leading to more robust and healthier plant growth^53^. A similar observation was made in blackgram^25^, greengram^54^ and okra^55^.

## Conclusions

This study conclusively demonstrates the effectiveness of botanical priming as a low-cost and eco-friendly technique to improve seedling attributes, hydrolytic enzymes, and yield significantly. Multiple lab and field studies suggest that botanicals can internalize seed coats and support water uptake inside seeds, accelerating various enzymatic activities and promoting seed germination. The study also indicated that priming with botanicals increases the root length due to the presence of phenol, which indirectly influences the germination % and increases the vigour index Ι and ΙΙ. Furthermore, as evidenced by biochemical activities, it can be hypothesized that the active ingredients in the botanicals, such as phenol and curcumin, accelerate the enzymes' activities, total proteins, and seed storage proteins. The low concentrations of botanicals help support early seedling establishment and prevent the attack of fusarium wilt disease during plant establishment, which leads to a healthy plant population and growth, ultimately resulting in higher crop yield. Therefore, botanical priming could be a cost-effective means of increasing the production and productivity of rainfed chickpea crops, which can further support the sustainable development of agriculture and improve the farmers' socio-economic condition. The study can be a boon for botanical priming applications for sustainable agricultural practices and the Agri-seed industry in the future.

## Material and methods

### Experimental materials

The present research used a medium-vigour seed lot of the desi type chickpea cv. PG-186. The seed lot that exhibited less than 85% germination was considered medium-vigour, and the standard germination test was conducted as per the International Seed Testing Association (ISTA) standard. Further, all the experiments and field study complies with local and national guidelines and regulations.

### Priming materials and techniques

Fresh turmeric and neem leaves were dry shaded for 5–6 days and then dried in a hot air oven for 2–3 h at 60 °C. Dried leaves were ground into fine powder using mixer grinder, and further dissolved in 100 mL distilled water, and left overnight at room temperature. Further, the solution was filtrated using Whatman filter paper no. 1. Hydropriming was carried out by soaking the seeds in distilled water. The seeds without priming and farmers' standard practice of 2 g/kg bavistin treatment were taken as control. Seeds were placed in petri dishes between moist filter paper for 18 h at (20 ± 1 °C) and (80–85%) relative humidity. They were then air-dried for 48 h to their original moisture content.

### Physiological analysis of chickpea upon priming

Physiological analysis was carried out of all the treatments, namely T_1_ (without priming), T_2_ (bavistin treatment), T_3_ (1% aqueous turmeric leaf extract), T_4_ (1% aqueous neem leaf extract), and T_5_ (hydropriming) by following completely randomized design. All the experiments were conducted in four replicates. Seeds of different treatments were placed in rolled paper towels and kept in a germination chamber at 20 ± 1 °C and 85% RH for a standard period of 8 days by following International Seed Testing Association (ISTA) protocols. All the physiological evaluations were carried out on the 8th day of germination.

### Seedling characteristic determination

The seeds were assessed for different morphological indexes of seedlings, such as germination percentage, root length (RL), shoot length (SL), seedling length (RL + SL), seedling dry weight, and seedling vigour indices I and II^56,57^. Once the dry weight of the seedlings was determined, ten of them were carefully wrapped in wax paper and kept in a hot air oven maintained at a precise temperature of 80 ± 2 °C for 17 h. After this, the seedlings were allowed to cool for 45 min in desiccators before being weighed using an electronic scale. The dry mass of each seedling was calculated and expressed in grams. Additionally, the germination rate (the percentage of average germinated seed out of all tested seeds at the end of the entire test) was calculated, and seedling vigour indices (seedling vigour index I and seedling vigour index II) were estimated using the standard formulae^57^. Seedling vigour index I determine the seeding vigour on the basis of seedling length which is calculated using germination (%) x seedling length (cm), while, seedling vigour index II determines the seeding vigour which is calculated on the basis of dry matter of seedling by using formula germination (%) x seedling dry weight (g).

### Assays of biochemical activities

Amylase activity was measured using Bernfeld's method^58^ with some modifications. 1 g of seed sample was ground in 10 ml of 10 mM CaCl_2_, and the supernatant was used for enzymatic activities. Starch and enzyme solutions were incubated at 27 °C for 30 min, and DNS reagent was added. After heating and adjusting the solution, optical density was measured at 560 nm, and a maltose standard curve was used. The enzymes were extracted from a 1 g seed sample in acetone and centrifuged to assess protease activity^59^. Add casein solution to each tube to assay enzyme activity and incubate at 35 °C. The enzyme solution was added and incubated again. TCA and sodium carbonate with FCR were added and incubated. The solution was filtered, and optical density was measured using a UV–Vis spectrophotometer at 600 nm. The dehydrogenase activity of primed and non-primed seed was quantified^60^, where imbibed seed samples of 200 mg were soaked in a freshly made, pH-7.0, 0.2% TTC solution in 10 ml. The solution was then incubated in the dark for 3 h at 30 °C. After draining the TTC solution, acetone was added to each tube for crushing, and the sample was incubated overnight before being centrifuged for 10 min at 10,000 rpm. Using a UV–Vis spectrophotometer, collect the supernatant and measure the absorbance at 480 nm.

The phytase activity was measured using a modified method^61^. One gram of seed sample was homogenized in 0.1 M sodium acetate buffer (pH 5.0) and centrifuged. Phytase activity was determined by adding buffer and sodium phytate solution, incubating, adding a crude enzyme, and incubating and measuring phosphate liberation with the ammonium molybdate method. MDA content was determined using 20% TCA and 0.5% TBA solution to quantify lipid peroxidation^62^. The seed sample was ground in 4 ml of 1% TCA solution. After centrifuging, the supernatant was collected. 1 ml of (20% TCA and 0.5% TBA) was added to each sample. The absorbance was accurately measured at 532 nm and 600 nm for specific and non-specific samples, following a rigorous incubation and centrifugation process. MDA content was measured in moles/ml.

### Calculation

$$\mathrm{MDA }\left({\text{mM}}\right)= \frac{{A}_{532}-{A}_{600}}{155}\times 100$$

The extinction coefficient of this MDA-TBA abduct at 532 nm is 155 mM^−1^ cm^−1^.

### Characterization of seed storage proteins

The total protein was estimated from chickpea seed, and a standard curve was produced using Bradford's standard solution^63^. A sample of dried chickpea seeds was ground using a hammer mill; fine powder was obtained, passed through a 0.185 mm mesh grid, and kept in air-tight plastic containers at room temperature to prevent spoilage. Chickpea flours were defatted overnight using a horizontal shaker with hexane in a ratio of 1:10 and then washed twice with ethyl ether, followed by drying for 1 h at − 20 °C. Protein fractions were obtained using the Osborne (1907) fractionation method^64^. Seed flours were extracted by stirring in borate buffer at pH-7.6 with NaCl and sodium azide for 2 h, followed by centrifugation at 30,000 rpm for 30 min.

### Polyacrylamide Gel Electrophoresis analysis

SDS-PAGE was carried out using the 5% stacking gel and 12% resolution gel^65^. Sample solutions were prepared from 10 mg of freeze-dried protein extract or precipitates dissolved in 1 ml sample buffer (distilled water, 0.5 M Tri-HCl pH 6.8, glycerol, 10% SDS, 1% bromophenol blue and β-mercaptoethanol heated at 98 °C for 10 min, then applied to the sample wells. Electrophoretic migration was monitored at a constant current (12 mA/gel) for 1.5 to 2 h. SDS gels were stored for two hours, and distaining was done for 12 h (Supplementary Information file 4).

### Field experiment

The field research trial used a randomized complete block design with eight replications and three priming treatments: T_1_ (dry seed as control), T_2_ (bavistin treated as positive control), and T_3_ (turmeric leaf extract primed). The seed was sown in 5 rows per plot, with a plot size of 2.8 × 1.8 m^2^. Field emergence percentage (no. of seedlings emerged/total no. of seed sown) was calculated 15 days and 30 days after sowing. Five randomly selected plants from each replicated plot of all treatments were tagged to take all the observations, such as plant height (at 15 DAS, 30 DAS, 45 DAS and maturity) and yield parameters. The biological yield was calculated before threshing by taking the total weight of harvested crop plants from the net plot area. The biological yield was given in kg ha^−1^. After sun drying for a few days, the harvested crop from the respective net plot was threshed with a thresher. Seed yield was recorded and expressed as kg/ha. The Harvest Index (HI) was calculated using formulae-

$$HI=\frac{Biological \;Yield}{Economic \;Yield}\times 100$$

### Statistical analysis

The collected data were analyzed using the statistical software SPSS and the analysis of variance technique. The treatment means were compared using a post-hoc test at a 5% significance level to determine whether there were any significant differences between them. Furthermore, a graphical representation of the data was created using Microsoft Excel to provide a clear and visual presentation of the results.

### Supplementary Information

Supplementary Information 1.

Supplementary Information 2.

Supplementary Information 3.

Supplementary Figures.

## Data Availability

All data generated or analysed during this study are included in this published article and its supplementary information files.
