# Supplementary material for: Physio-biochemical responses and crop performance analysis in chickpea upon botanical priming
Source: Sci Rep. 2024 Apr 23;14:9342. doi: 10.1038/s41598-024-59878-8 (PMC11039450; doi:10.1038/s41598-024-59878-8)
Supplement: Supplementary file 4 — Supplementary Figures. [file 41598_2024_59878_MOESM4_ESM.docx]

**12 hr Turmeric Priming**

**Bavisitin**

**18 hr Hydro Priming**

**12 hr Hydro Priming**

**18 hr Neem Priming**

**12 hrNeem Priming**

**18 hr Turmeric Priming**

**Non primed**


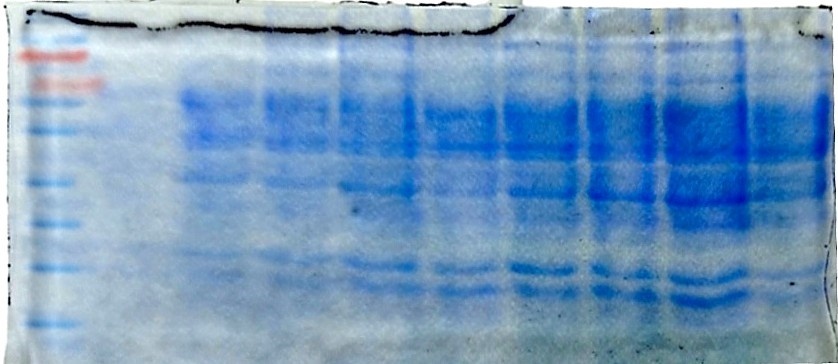


**Fig. S1:** Original SDS-PAGE image used in Fig. 7 (control, 12 & 18 hr turmeric/neem/hydro priming).

**6 hr Hydro Priming**

**6 hr Neem Priming**

**6 hr Turmeric Priming**

**
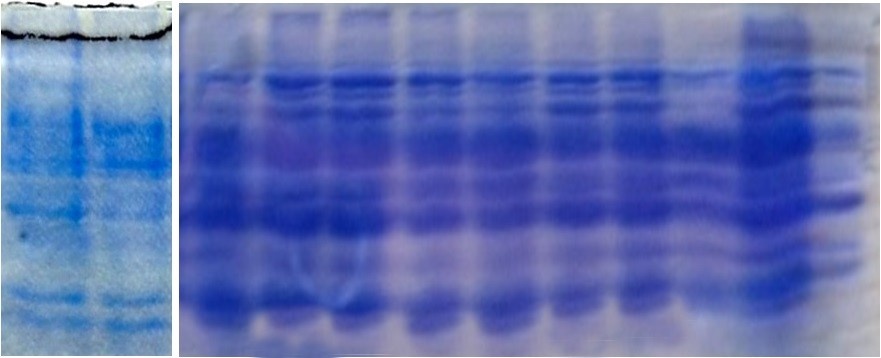
**

**Fig. S2:** Original SDS-PAGE image used in Fig. 7 (6 hr neem priming).
